# Parental Education and Children Ever Born Among Women in Bangladesh: Evidence From a Nationally Representative Survey

**DOI:** 10.1002/puh2.70366

**Published:** 2026-09-10

**Authors:** Farhia Azrin, Md. Muddasir Hossain Akib, Md. Salek Miah, Tanzila Tamanna, Bikash Pal, Haroon Bin Murshid, Margia Yesmin

**Affiliations:** ^1^ Institute of Statistical Research and Training University of Dhaka Dhaka Bangladesh; ^2^ Department of Statistics University of Dhaka Dhaka Bangladesh; ^3^ Department of Statistics Shahjalal University of Science and Technology (SUST) Sylhet Bangladesh; ^4^ Department of Statistics and Data Science Jahangirnagar University Dhaka Bangladesh; ^5^ Faculty of Medicine University of Dhaka Dhaka Bangladesh; ^6^ Department of Mathematics and Statistics Bangladesh University of Textiles Dhaka Bangladesh

**Keywords:** Bangladesh Demographic and Health Survey, Conway–Maxwell–Poisson regression model, fertility, parental education, underdispersion

## Abstract

**Background:**

Fertility is a key determinant of population growth and overall population size. Bangladesh's total fertility rate has declined from about 6.3 births per woman in the mid‐1970s to 2.3 in 2022, which is around the replacement level and, by some recent estimates, slightly below it. Yet the country remains among the world's most densely populated, and population momentum will sustain growth for decades. Understanding how parental education is associated with fertility, therefore, remains crucial for population and reproductive health policy.

**Methods:**

This cross‐sectional study analyzed 3839 ever‐married women aged 15–49 years from the nationally representative Bangladesh Demographic and Health Survey (BDHS) 2022. The outcome was the number of children ever born (CEB). Because the counts were underdispersed, a mean‐parameterized Conway–Maxwell–Poisson regression model, selected over the Poisson and generalized Poisson models by Akaike Information Criteria (AIC) and Bayesian Information Criteria (BIC), was fitted with BDHS sampling weights, adjusting for relevant sociodemographic characteristics.

**Results:**

Maternal education showed a strong, graded inverse association with CEB. Compared with women with no education, mothers with primary (incidence rate ratio [IRR]: 0.95; 95% CI: 0.91–0.99), secondary (IRR: 0.87; 95% CI: 0.83–0.91), and higher education (IRR: 0.72; 95% CI: 0.68–0.77) had significantly fewer CEB. Paternal education was similarly associated with lower fertility: Compared with fathers with no education, those with secondary (IRR: 0.94; 95% CI: 0.91–0.97) and higher education (IRR: 0.92; 95% CI: 0.88–0.97) had fewer children. Region, residence, wealth, maternal employment, religion, and desire for more children were also significantly associated with CEB.

**Conclusions:**

Parental education, particularly maternal education, is strongly and consistently associated with lower fertility in Bangladesh. Although the cross‐sectional design precludes causal inference, the findings suggest that strengthening educational opportunities, expanding access to reproductive health information and family planning services, and addressing socioeconomic disparities may help consolidate Bangladesh's fertility transition.

## Graphical abstract

Using nationally representative BDHS 2022 data, higher maternal and paternal education were independently associated with fewer children ever born among Bangladeshi women. The strongest association was observed for higher maternal education (28% fewer children), highlighting education as a key driver of fertility transition and reproductive health policy.

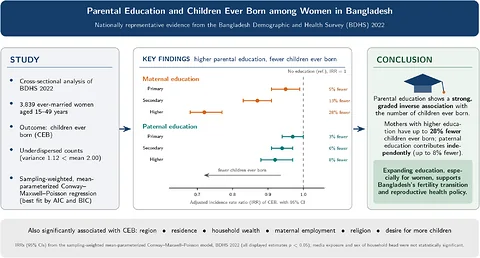

## 1 Introduction

The world's population has more than tripled since 1950 and, according to the United Nations, is projected to continue growing until the mid‐2080s [1]. Fertility is the principal proximate driver of this growth, as it plays a vital role in structuring population size [2]. Although the global total fertility rate (TFR) has declined from about five children per woman in the 1960s to approximately 2.3 at present, births remain concentrated in low‐ and lower middle‐income countries, where continued rapid growth strains health, education, and social protection systems [1, 3, 4].

Children ever born (CEB) is widely used in developing countries to understand fertility dynamics, as it is considered a major indicator of fertility [2, 5]. CEB refers to the total number of live births a woman has had, regardless of whether the children are still alive, and excludes stillbirths, abortions, and adopted children [5, 6]. CEB data across age groups can reflect overall childbearing trends and are useful for assessing fertility patterns where reliable vital registration is lacking [3]. The average number of CEB per woman, therefore, bears directly on healthcare planning, economic policymaking, and resource allocation [7].

Bangladesh, one of the most densely populated countries in the world, had an estimated population of about 176 million in 2025, which is projected to exceed 200 million around mid‐century [1]. At the same time, Bangladesh has achieved one of the most remarkable fertility transitions in the developing world: The TFR declined from about 6.3 births per woman in 1975 to 2.3 in 2022, and recent United Nations estimates place it at, or slightly below, the replacement level of 2.1 [1, 8]. This decline has been attributed primarily to the expansion of female education and the national family planning program, alongside rising contraceptive use, later marriage, and broader socioeconomic development [9, 10, 11]. The government continues to promote the small‐family norm with the slogan “Not more than two children; one is better” [12]. Even so, because of population momentum and the country's exceptional density, the absolute population will keep growing for decades, and sustaining low fertility remains a stated target of the fourth Health, Population, and Nutrition Sector Program (HPNSP) [13]. Understanding the determinants of childbearing, therefore, remains a policy priority.

The analysis of fertility decline rests on a substantial theoretical literature. Classical demographic transition theory interprets sustained fertility decline as a response to modernization, urbanization, and falling mortality [14, 15]. In Becker's quantity–quality framework, rising parental education, especially maternal education, increases the opportunity cost of childbearing and shifts parental investment from the number of children toward the education and health of each child [16]. Caldwell's wealth‐flows theory emphasizes that mass schooling reverses the direction of intergenerational transfers, making children economically costly rather than beneficial and thereby eroding the rationale for large families [14]. Bongaarts’ proximate‐determinants framework clarifies that socioeconomic variables, such as education, influence fertility only through behavioral channels, chiefly marriage timing, contraceptive use, breastfeeding, and abortion [17]. Ideational and diffusion perspectives add that education accelerates the spread of small‐family norms and strengthens women's autonomy in reproductive decision‐making [18]. Together, these perspectives predict an inverse association between education and fertility that is strongest for women's schooling; the present study examines that prediction for Bangladesh.

A large body of empirical evidence supports this prediction: Education, particularly women's education, is consistently associated with lower fertility [19, 20]. Educated women are more aware of their health and family planning options, which strengthens their say in decisions about childbearing [21], and education enables women to postpone marriage, participate in the labor force, and invest more intensively in each child [20, 22]. In Bangladesh, recent studies have revisited the drivers of the fertility transition: Bora et al. found that female education and the family planning program were the leading contributors to the country's fertility decline [9], whereas Maitra highlighted the roles of globalization and economic and social development [10]. Count‐data analyses of CEB in Bangladesh have applied zero‐truncated, Bayesian, and generalized Poisson (GP) models [5, 13, 23]. Nevertheless, important gaps remain. Most existing studies focus on women's education alone, and the role of paternal education has received comparatively little attention, even though fathers are usually the principal decision‐makers in Bangladeshi households, and related Bangladesh Demographic and Health Survey (BDHS)‐based work suggests that parental education shapes other child‐related outcomes [24]. In addition, few studies have examined the most recent BDHS 2022 data, and fewer still have used count models appropriate for underdispersed outcomes.

The number of children is counting data, and this type of data is usually handled by the Poisson regression model (PRM) [23, 24, 25]. PRM holds the assumption that the mean and variance of the count variable are equal [26, 27]. Although Poisson regression is commonly used as a baseline model for analyzing count data under the assumption of equidispersion, real‐world count data frequently exhibit either overdispersion or underdispersion, violating this assumption [28, 29]. The dispersed nature of count data in modeling may provide misleading interpretations if ignored [30, 31]. In particular, the widely used negative binomial model accommodates only overdispersion, whereas the GP and Conway–Maxwell–Poisson (CoMPois) families can accommodate underdispersion as well [32, 33, 34, 35]. Accordingly, this study compares candidate count models and bases inference on the specification best supported by the data.

Against this backdrop, the objective of this study is to examine the association between parental education, both maternal and paternal, and the number of CEB among ever‐married women in Bangladesh, using the nationally representative BDHS 2022 data [8] and adjusting for sociodemographic characteristics. The study contributes to the existing literature in three ways. First, it considers maternal and paternal education jointly, rather than women's schooling alone. Second, it provides up‐to‐date evidence from the most recent (2022) round of the BDHS. Third, it accounts explicitly for the underdispersion of the CEB counts by employing a mean‐parameterized Conway–Maxwell–PRM, thereby illustrating a modeling strategy that is appropriate when standard Poisson and negative binomial approaches are not. The findings are intended to inform education, family planning, and population policies in Bangladesh and in comparable low‐ and middle‐income settings.

## 2 Materials and Methods

### 2.1 Data Source

This article focused on the BDHS, 2022 dataset for analysis [8]. BDHS is a part of the Demographic and Health Survey (DHS) program by the United States of America (USA) and represents Bangladesh's national scenario. This is a cross‐sectional survey conducted in two separate stages using a stratified cluster sampling design [8]. In the earlier stage, 675 enumeration areas (EAs) were selected, where EAs were selected with probability proportional to size. After that, 45 households from each EA were systematically selected for interview. All ever‐married women aged between 15 and 49 years who were either usual residents of the selected households or stayed in the selected households the night before the survey were eligible for the interview. After removing all the missing cases, we ended up with a dataset, including the information of 3839 respondents. Consistent with the BDHS sampling frame, the analytic sample is restricted to ever‐married women aged 15–49 years; the implications of this restriction for interpretation are considered in Section 4. To account for the unequal selection probabilities arising from the complex survey design, the BDHS sampling weights were applied in the estimation of all regression models, and the survey's stratification variables (administrative division and urban–rural residence) were included as covariates. Clustering at the level of the primary sampling units and the stratification of the design were not otherwise incorporated into variance estimation; this is acknowledged in Section 6.

### 2.2 Patients Consent Statements

This study does not include any patients.

### 2.3 Variables

This study's response variable of interest is the number of CEB, defined as the total number of live births a woman has ever had, irrespective of whether the children were still alive at the time of the survey [5, 6]; for consistency, the outcome is referred to as CEB throughout the text and tables. This study considers various socioeconomic and demographic factors as explanatory variables. The covariates, namely, are region (Dhaka, Barishal, Chittagong, Khulna, Mymensingh, Rajshahi, Rangpur, and Sylhet) which are administrative divisions in Bangladesh, type of residence (rural and urban), mother's education level (no education, primary, secondary, and higher), father's educational level (no education, primary, secondary, and higher), mother's current age at the time of the survey, in years (<20, 20–35, and >35), wealth index (poor, middle, and rich; derived using PCA on the basis of ownership of household goods), mother's working status (no and yes), exposure to media (exposed and unexposed), sex of household head (female and male), mother's religion (Muslim and others), and desire for more children (no and yes). Some variables and their categories were not directly available in the survey dataset, so they were recategorized in this study. For example, a mother is classified as being exposed to the media if she reads newspapers or magazines, listens to the radio, or watches television at least once a week [22, 36, 37, 38, 39]. Households were originally categorized into five wealth quintiles (poorest, poorer, middle, richer, and richest). For the purposes of this study, these quintiles were consolidated into three groups: poor (poorest and poorer), middle, and rich (richer and richest) [40, 41, 42]. The desire for more children was included to account for a woman's stage of family building and her fertility preferences. Because current parity can itself influence whether a woman wants additional children, this covariate is potentially endogenous to the outcome; it is therefore treated as an adjustment variable, its coefficient is interpreted descriptively rather than causally, and this issue is revisited in Section 6.

### 2.4 Models

Three different regression models were considered to analyze the data: Poisson, GP, and Conway–Maxwell–Poisson. Initially, we considered Poisson regression, as it is a parametric benchmark for count data [43, 44]. Count data cannot be illustrated by linear regression, as it violates the assumptions of continuity and normality [28]. The PRM assumes equidispersion, meaning the mean and variance are equal. It utilizes the log link; as a result, the model is linear in terms of the logarithm of the count data. Hence, we applied the PRM in a generalized modeling framework to analyze the number of CEB in Bangladesh. The probability mass function of Poisson regression is as follows:

$$f\left(Y_{i},=y_{i},\;|\;xi\right)=\frac{\left(e^{-\mu_{i}}\times {\mu_{i}}^{yi}\right)}{y_{i}!};\;y_{i}=0,1,2,\text{…},\;\mathrm{where}\;\mu_{i}=e^{{\mathrm{xi}}^{\prime }\beta }$$

However, in practical scenarios, primarily the data do not show equidispersion, meaning the mean and variance are not equal [24]. Therefore, different options are considered when modeling the data. Another suitable option for modeling count data is the GP, which can deal with both overdispersion and underdispersion, although the range of dispersion it can accommodate is limited [33, 34]. By considering some parametric transformation, the following PMF is followed by GP [33]:

$$f\left(y_{i},\mu_{i},\alpha \right)={\left(\frac{\mu_{i}}{1+\alpha \mu_{i}}\right)}^{y_{i}}\frac{\left(1+\alpha y_{i}\right)}{y_{i}!}\exp\left[\frac{-\mu_{i}\left(1+\alpha y_{i}\right)}{1+\alpha \mu_{i}}\right];y_{i}=0,1,2,\cdots$$

with $E\left(Y_{i}\right)=\mu_{i}\;\mathrm{and}\;\mathrm{Var}\left(Y_{i}\right)=\mu_{i}{\left(1+\alpha \mu_{i}\right)}^{2}$. If α = 0, the GP turns into Poisson, for α > 0 and α < 0, it reflects overdispersion and underdispersion, respectively. To overcome the limitation of GP, another suitable option to model count data is CoMPois, which can deal with both underdispersion and overdispersion [45, 46]. The probability mass function of the CoMPois model is

$$\begin{array}{ccc} P\left(\;Y_{i}=y_{i}|\lambda ,v\right) & = & \frac{\lambda^{v}}{{\left(y_{i}!\right)}^{v}Z\left(\lambda ,v\right)},\;y=0,1,2,\text{…},n,\;\mathrm{where}\;z\left(\lambda ,v\right)\\ & & =\sum_{s=0}^{\infty }\frac{\lambda^{s}}{{\left(s!\right)}^{v}},\;\lambda >1,\;v\geq 0\; \end{array}$$

with $E\left(Y_{i}\right)=\mu_{i}\approx \lambda^{1/v}-\frac{v-1}{2v}$ and $\mathrm{Var}\left(Y_{i}\right)\approx \frac{1}{v}\lambda^{1/v}$. Here, z(λ,v) is the normalization constant, v>1 indicates underdispersion, and v<1 indicates overdispersion. However, CoMPois has a major drawback, which is that it cannot model the mean directly. To solve the issue associated with CoMPois, we used the mean‐parameterized CoMPois as it allows us to model the mean counts [44]. The model can be used by maximizing the log‐likelihood $\log\;L\left(\theta ,v\right)=\sum_{i=1}^{n}\left(y_{i}\;\log\lambda \left(\mu_{i},v\right)-v\;\log\left(y_{i}!\right)\;\log\;z\left(\lambda (\mu_{i},v),v\right)\right)$ [44]. Here, the link function is $E\left(Y_{i}\right)=\mu_{i}=\exp\left(x_{i}^{⊤}\beta \right)$, where the vector of explanatory variables is $x_{i}={\left(x_{i1},\text{…},x_{ij},\text{…},x_{ip}\right)}^{\prime },$ and the vector of regression coefficients is $\beta ={\left(\beta_{i1},\text{…},\beta_{ij},\text{…},\beta_{ip}\right)}^{\prime }$ and the parameters $\theta ={\left(\beta ,\;v\right)}^{\prime }.$

The negative binomial regression model, although the most common alternative to the Poisson model for dispersed counts, was deliberately not considered, because its variance function, Var(*Y_i_*) = *µ_i_* + *αµ_i_* ^2^ with *α* > 0, can accommodate only overdispersion, that is, a variance exceeding the mean [32]. As shown in Table 1, the number of CEB in our sample is underdispersed (variance 1.12 vs. mean 2.00; dispersion ratio 0.56), a situation for which the GP and CoMPois families are the appropriate choices [33, 35, 44]. The adequacy of the selected model was assessed by comparing the information criteria of the candidate models (Table 2), and the direction of dispersion implied by the fitted model was consistent with the underdispersion observed in the raw counts.

**TABLE 1:** Descriptive statistics for the number of children ever born.

Characteristics	Number of children ever born
Sample size	3839
Mean	2.00
Variance	1.12

**TABLE 2:** Selection of model on the basis of Akaike Information Criteria (AIC) and Bayesian Information Criteria (BIC) for the number of children ever born.

AIC			BIC		
PRM	GPRM	CMPRM	PRM	GPRM	CMPRM
10,668.8	8383.7	7642.1	10,818.8	8540.0	7798.4

Abbreviations: CMPRM, Conway–Maxwell–Poisson regression model; GPRM, generalized Poisson regression model; PRM, Poisson regression model.

To determine the best‐fitting model among these available options, Akaike Information Criteria (AIC) and Bayesian Information Criteria (BIC) were used. The model was considered the best fit when it had the minimum AIC and BIC values [47]. For a model with p parameters and n observations, AIC and BIC can be represented as $\mathrm{AIC}=-2l(\hat{\theta },y)+2p$, $\mathrm{BIC}=-2l\left(\hat{\theta },y\right)+p\;\log\left(n\right)$, where $\;l(\hat{\theta },y)$ represents the log‐likelihood. The models were estimated by maximum likelihood using the glmmTMB package [48] in R version 4.3.3, with the BDHS sampling weights applied in estimation; descriptive analyses were performed using IBM SPSS Statistics version 27.

## 3 Results

Table 3 represents socioeconomic and demographic characteristics of women on the basis of their number of ever‐born children, where region, residence, educational qualification of mother, mother's age, father's educational qualification, wealth index, mother's working status, media exposure, religion, desire for more children, and sex of household head were explored. According to the findings, in the group of mothers with no education, the number of mothers having more children increased, and it is visible that only 17.02% had one child, and 28.70% of mothers had three children, whereas the scenario is opposite with mothers with higher education. Similarly, only 21.79% fathers with no education had one child. In comparison, 31.77% had two children, and 26.53% had three children, whereas the scenario is opposite for fathers with higher education, where 49.41% had one child, and only 9.8% had three children. It reflects that mothers from Dhaka (41.42%) and Rangpur (41.71%) divisions tended to have one child, and the percentage of having more children decreased. A large proportion of middle‐aged mothers (44.98%) preferred having two children, whereas older mothers tended to have three (28.25%) or four (27.36%) children. Mothers from wealthy backgrounds mostly had one or two children (39.34% and 38.07%), whereas more than one‐fourth (26.91%) of poorer mothers had three or four children. Working mothers had a higher concentration of having two children (40.74%), whereas nonworking mothers had a higher concentration of having one child (39.98%). Media exposure also plays a prominent role, where exposed mothers were more prone (40.02%) to have two children, and unexposed mothers were more likely (35.62%) to have one child, although more than one‐fifth (20.48%) of unexposed mothers had three children. Mothers from other religions mostly had one or two children (40.22% and 43.09%), whereas the percentage of having one or two children was 36.10% and 35.81% in Muslim mothers. Women who were the head of the house showed a greater tendency to have children, and more than one‐fourth (25.58%) of mothers had three children. On the other hand, the percentage of having three children was only 17.44% in male‐led families.

**TABLE 3:** Sociodemographic characteristics of women by row percentage on children ever born.

Variable	Number of children ever born									
	0	1	2	3	4	5	6	7	8	9
**Mother's educational level**										
No education	0	17.02	26.57	28.7	14.82	6.17	4.12	0.64	1.23	0.72
Primary	0.43	23.89	32.65	27.56	11.27	3.27	0.25	0.54	0.14	0
Secondary	0.8	39.05	37.75	16.7	4.35	1.23	0.12	0	0	0
Higher	0.56	50.43	40.43	8.28	0.19	0	0	0.1	0	0
**Father's educational level**										
No education	0.85	21.79	31.77	26.53	11.71	4.36	1.62	0.46	0.66	0.26
Primary	0.55	32.35	36.27	20.81	7.67	1.9	0.25	0.18	0	0
Secondary	0.76	39.21	37.8	17.26	3.35	1.39	0.06	0.16	0	0
Higher	0.34	49.41	37.99	9.8	2.39	0.07	0	0	0	0
**Region**										
Dhaka	0.22	41.42	36.31	16.63	3.98	1.23	0	0	0.21	0
Barishal	0.74	29.32	42.51	19.98	5.34	2.11	0	0	0	0
Chittagong	0.25	35.61	31.26	22.65	6.3	2.27	0.96	0.45	0	0.24
Khulna	0.84	38.13	41.3	15.18	3.43	0.78	0	0.13	0.2	0
Mymensingh	0	32.68	36.32	17.22	8.4	3.94	0.46	0.63	0.34	0
Rajshahi	1.41	36.86	43.28	15.76	2.69	0	0	0	0	0
Rangpur	0.67	41.71	33.14	16.57	6.21	1.46	0.24	0	0	0
Sylhet	1.33	34.9	31.6	20.77	8.92	1.57	0.81	0.1	0	0
**Residence**										
Rural	0.54	35.46	35.55	19.37	6.2	2.05	0.39	0.27	0.11	0.06
Urban	0.8	38.53	38.33	16.11	4.81	1.09	0.25	0	0.07	0
**Mother's age (years)**										
<20	1.13	85.84	12.88	0.16	0	0	0	0	0	0
20–35	0.48	23.68	44.98	23.17	5.91	1.48	0.17	0.11	0	0
>35	0.51	2.98	20.16	28.25	27.36	12.34	4.01	1.85	1.82	0.71
**Wealth index**										
Middle	0.43	41.46	32.11	18.83	5.89	0.86	0.36	0.06	0	0
Poor	0.88	31.21	37.02	19.31	7.6	2.83	0.57	0.31	0.19	0.1
Rich	0.48	39.34	38.07	17	3.79	1.06	0.11	0.1	0.06	0
**Currently working**										
No	0.54	39.98	35.24	17.55	4.84	1.39	0.26	0.15	0	0.05
Yes	0.93	24.19	40.74	20.9	8.91	2.97	0.65	0.28	0.44	0
**Media exposure**										
Unexposed	0.6	35.62	31.87	20.48	7.7	2.53	0.6	0.27	0.22	0.09
Exposed	0.65	37.12	40.02	16.61	4.23	1.12	0.14	0.11	0	0
**Religion**										
Others	0.54	40.22	43.09	11.48	4.31	0.35	0	0	0	0
Muslim	0.64	36.1	35.81	18.97	5.89	1.87	0.38	0.2	0.11	0.04
**Desire for more children**										
No	0.14	10.23	47.11	29.02	9.54	2.93	0.49	0.31	0.17	0.07
Yes	1.3	72.5	21.85	3.57	0.54	0.1	0.14	0	0	0
**Sex of household head**										
Female	0.07	33.3	34.55	25.58	5.52	0.98	0	0	0	0
Male	0.69	36.84	36.69	17.44	5.77	1.83	0.38	0.2	0.11	0.04

Table 1 reflects the mean and variance of the number of CEB, where the variance (1.12) is smaller than the mean (2.00), suggestive of underdispersion (dispersion ratio = 0.56). As a result, we have explored the generalized PRM (GPRM) and the Conway–Maxwell PRM (CMPRM) alongside the PRM to model the number of CEB.

Table 2 shows the AIC and BIC values to assess the performance of three different models, PRM, GPRM, and CMPRM, broadly used to analyze count data. The AIC values for PRM, GPRM, and CMPRM are 10,668.8, 8383.7, and 7642.1, respectively, and the BIC values reflect the same pattern, showing 10,818.8, 8540.0, and 7798.4 values for the above‐mentioned model, respectively. The CMPRM model performed better in both the AIC and BIC metrics, as lower AIC and BIC indicate better fit. Therefore, for further analysis and discussion, this study has demonstrated results produced by the CMPRM model.

Table 4 presents the adjusted associations between socioeconomic and demographic factors and the number of CEB estimated from the CMPRM. According to the table, region, residence, mother's educational qualification, mother's age, father's educational qualification, wealth index, mother's employment status, religion, and desire for more children were significantly associated with the number of CEB, whereas media exposure (*p* = 0.065) and sex of household head (*p* = 0.105) were not statistically significant at the 5% level. Mothers’ educational qualifications were one of the strongest factors: The rate of CEB was 5% (incidence rate ratio [IRR]: 0.95, 95% CI: 0.91–0.99, *p* < 0.05), 13% (IRR: 0.87, 95% CI: 0.83–0.91, *p* < 0.001), and 28% (IRR: 0.72, 95% CI: 0.68–0.77, *p* < 0.001) lower among mothers with primary, secondary, and higher education, respectively, compared with uneducated mothers. The pattern was similar for educated fathers: Compared with fathers with no education, the rate of CEB was 3% (IRR: 0.97, 95% CI: 0.94–1.00, *p* < 0.05), 6% (IRR: 0.94, 95% CI: 0.91–0.97, *p* < 0.01), and 8% (IRR: 0.92, 95% CI: 0.88–0.97, *p* < 0.01) lower among fathers with primary, secondary, and higher education, respectively. There were also significant regional differences: Compared with Dhaka, the rate of CEB was 13% higher in Chittagong (IRR: 1.13, 95% CI: 1.08–1.17, *p* < 0.001), 8% higher in Mymensingh (IRR: 1.08, 95% CI: 1.04–1.12, *p* < 0.001), and 7% higher in Sylhet (IRR: 1.07, 95% CI: 1.03–1.11, *p* < 0.01). The rate of CEB was 5% lower among urban mothers than among rural mothers (IRR: 0.95, 95% CI: 0.92–0.97, *p* < 0.001). Relative to middle‐wealth households, both poor (IRR: 1.03, 95% CI: 1.00–1.07, *p* = 0.031) and rich (IRR: 1.04, 95% CI: 1.01–1.07, *p* = 0.016) households showed slightly higher rates of CEB. Working mothers had an 8% higher rate of CEB than nonworking mothers (IRR: 1.08, 95% CI: 1.05–1.11, *p* < 0.001), and Muslim mothers had a 14% higher rate than mothers of other religions (IRR: 1.14, 95% CI: 1.11–1.19, *p* < 0.001). Finally, women who desired more children had a 37% lower number of CEB to date than those who did not (IRR: 0.63, 95% CI: 0.62–0.65, *p* < 0.001), a pattern that largely reflects their earlier stage of family building.

**TABLE 4:** Incidence rate ratio (IRR) on children ever born (CEB), with *p* values and CIs, from the CMPRM.

Covariate	IRR	Lower CI	Upper CI	*p* value
**Mother's educational level**				
No education				
Primary	0.95	0.91	0.99	0.013
Secondary	0.87	0.83	0.91	**<0.001**
Higher	0.72	0.68	0.77	**<0.001**
**Father's educational level**				
No education				
Primary	0.97	0.94	1	0.027
Secondary	0.94	0.91	0.97	0.001
Higher	0.92	0.88	0.97	0.001
**Region**				
Dhaka				
Barishal	1.03	0.99	1.08	0.126
Chittagong	1.13	1.08	1.17	**<0.001**
Khulna	0.97	0.93	1.02	0.230
Mymensingh	1.08	1.04	1.12	**<0.001**
Rajshahi	0.96	0.92	1.01	0.107
Rangpur	1	0.96	1.04	0.987
Sylhet	1.07	1.03	1.11	0.002
**Residence**				
Rural				
Urban	0.95	0.92	0.97	**<0.001**
**Mother's age (years)**				
<20				
20–35	1.59	1.53	1.65	**<0.001**
>35	2.21	2.1	2.32	**<0.001**
**Wealth index**				
Middle				
Poor	1.03	1	1.07	0.031
Rich	1.04	1.01	1.07	0.016
**Current working status**				
Unemployed				
Employed	1.08	1.05	1.1	**<0.001**
**Media exposure**				
Unexposed				
Exposed	0.98	0.96	1	0.065
**Religion**				
Others				
Muslim	1.14	1.1	1.19	**<0.001**
**Desire for more children**				
No				
Yes	0.63	0.62	0.65	**<0.001**
**Sex of household head**				
Female				
Male	1.03	0.99	1.06	0.105

*Note:* Reference categories are shown without estimates. IRRs and confidence limits are rounded to two decimal places; confidence limits displayed as 1.00 result from rounding, and the exact limits exclude unity, consistent with the corresponding *p* values (e.g., father's primary education, *p* = 0.027; poor wealth index, *p* = 0.031). Bold font indicates *p* values <0.001.

Abbreviations: CI = confidence interval; CMPRM = Conway–Maxwell–Poisson regression model; IRR = incidence rate ratio.

## 4 Discussion

Using nationally representative BDHS 2022 data and a count model suited to underdispersed outcomes, this study found a strong, graded inverse association between parental education and the number of CEB in Bangladesh. These findings accord closely with theoretical expectations. The steep maternal‐education gradient is consistent with the quantity–quality framework, in which schooling raises the opportunity cost of childbearing and shifts parental investment toward child “quality” [16], and with wealth‐flows theory, which links mass education to the erosion of the economic rationale for large families [14]. They also align with recent decomposition evidence for Bangladesh showing that female education, together with the family planning program, has been the leading driver of the country's fertility decline [9], and with comparative evidence from low‐ and middle‐income countries [49]. Plausible mechanisms include better knowledge and use of contraception, later marriage, greater female autonomy in reproductive decision‐making, and stronger labor‐force attachment [20, 21]; we emphasize, however, that these mechanisms are interpretations informed by theory and prior literature rather than pathways tested directly in our data.

The independent association between paternal education and lower CEB, although smaller than the maternal gradient, is noteworthy in a context where fathers are usually the principal household decision‐makers. Educated fathers may be more receptive to family planning, more conscious of the costs of child‐rearing, and more likely to communicate and decide jointly with their spouses on fertility matters. These interpretations are plausible but were not directly tested here; establishing the behavioral channels through which paternal education operates would require data on couples’ communication, contraceptive decision‐making, and gender attitudes.

Regarding other socioeconomic factors, mothers residing in urban areas had significantly fewer CEB than rural mothers. This is consistent with the well‐documented urban advantage in access to healthcare and family planning services [50, 51, 52, 53], with the higher costs of living and child‐rearing, later marriage, and stronger career orientation typically found in urban settings, and with broader evidence that socioeconomic development underpins fertility decline in Bangladesh [10].

The association between household wealth and CEB was small and non‐monotonic: Relative to middle‐wealth households, both poor and rich households had slightly higher rates of CEB. Although the finding for wealthier households may seem counterintuitive from the standpoint of high‐income countries, financial capacity may ease the affordability constraint on additional children, and wealth in Bangladesh may partly support traditional extended‐family structures [54]. These explanations remain speculative, and the small magnitudes of the estimates (IRRs of 1.03–1.04) caution against strong substantive interpretation.

Employed mothers had a modestly higher number of CEB than non‐employed mothers. In Bangladesh, women are predominantly employed in informal sectors that can be more compatible with childbearing and traditional family responsibilities [55]. It is also possible that the direction of influence runs partly the other way: Women with larger families may enter income‐generating work out of economic necessity. This association should therefore not be read causally.

Family size also differed by religion, with Muslim mothers having a higher rate of CEB than mothers of other religions. This may partly reflect differences in religiosity, family‐size norms, and marriage patterns [20]; however, we did not measure religious attitudes or practice directly, and this association should be interpreted as descriptive.

In contrast, media exposure and the sex of the household head were not significantly associated with CEB at the conventional 5% level (*p* = 0.065 and *p* = 0.105, respectively). The directions of these estimates (slightly fewer children among media‐exposed mothers and slightly more in male‐headed households) are at most suggestive, and we refrain from drawing substantive conclusions from them.

The strong negative association between the desire for more children and CEB deserves a specific caution. Because women earlier in the family‐building process have both fewer births to date and a greater desire for additional children, this association is largely mechanical and may also reflect reverse causality, as current parity directly shapes the desire for more children. The variable was retained as an adjustment for a woman's stage of family building rather than as a substantive predictor, and its coefficient should not be interpreted causally.

Two features of the analytic sample also condition the interpretation of the findings. First, in line with the BDHS design, the sample includes only ever‐married women. Because education reduces fertility partly by delaying marriage [22], estimates conditioned on marriage capture post‐marriage fertility differentials and may understate the total association between education and lifetime fertility that operates through delayed or forgone marriage; the findings likewise generalize to ever‐married women rather than to all women. Second, the sample spans ages 15–49, so CEB reflects childbearing to date rather than completed fertility, and a young woman's CEB is not comparable to that of a woman nearing the end of her reproductive years. The regression models adjust for age group, and the age‐specific distributions in Table 3 behave as expected, but analyses restricted to women closer to completed fertility (e.g., ages 40–49) would be a valuable extension.

Overall, this study finds parental education to be a strong and consistent correlate of lower fertility in Bangladesh. Strengthening educational opportunities for both women and men, expanding access to family planning services, and promoting reproductive health education remain promising policy directions for consolidating the country's fertility transition, alongside continued attention to regional and socioeconomic disparities.

## 5 Conclusion

This study examined how parental education is associated with the number of CEB in Bangladesh using BDHS 2022 data and a Conway–Maxwell–PRM appropriate for the underdispersed outcome. We found a strong, graded inverse association between both maternal and paternal education and CEB, alongside significant associations with region, residence, wealth, maternal employment, religion, and fertility desire. These findings are consistent with the central role that fertility theory assigns to education, particularly women's education, and support continued investment in parental education as a key component of population policy. Efforts to promote reproductive health education, expand family planning services, and ensure equitable access to schooling remain crucial for sustaining Bangladesh's fertility transition. Given the cross‐sectional design, these results describe associations rather than causal effects, and future research using longitudinal or quasi‐experimental designs, couple‐level data, and samples of women with completed fertility would help clarify the causal pathways involved.

## 6 Limitations

This study has several limitations. First, BDHS 2022 is a cross‐sectional survey, which precludes causal inference; all reported estimates should be interpreted as associations. Second, although a wide range of socioeconomic and demographic factors was considered, unobserved confounders, such as couples’ fertility preferences at marriage, family background, and community‐level family planning supply, may influence the results. Third, the desire for more children is potentially endogenous to the outcome, because current parity affects whether a woman wants additional children; it was retained only as an adjustment for the stage of family building, and its estimate should not be interpreted causally. Fourth, although the BDHS sampling weights were applied in estimation, and the stratification variables (division and urban–rural residence) were included as covariates, clustering within primary sampling units and the stratification of the survey design were not incorporated into variance estimation (e.g., through cluster‐robust or design‐based standard errors); standard errors may, therefore, be somewhat understated. Fifth, the sample is restricted to ever‐married women aged 15–49, so the findings do not capture the pathway from education to fertility that operates through delayed or forgone marriage, and CEB for younger women reflects incomplete rather than completed fertility. Finally, some variables were recategorized for analysis, which may introduce classification bias, and birth histories are self‐reported and subject to recall error.

## Author Contributions

**Farhia Azrin**: writing – original draft. **Md. Muddasir Hossain Akib**: conceptualization, methodology, writing – review and editing. **Md. Salek Miah**: writing – review and editing. **Tanzila Tamanna**: writing – review and editing. **Bikash Pal**: writing – review and editing. **Haroon Bin Murshid**: formal analysis. **Margia Yesmin**: supervision.

## Funding

The authors have nothing to report.

## Ethics Statement

The authors have nothing to report.

## Conflicts of Interest

The authors declare no conflicts of interest.

## Data Availability

The datasets analyzed during the current study are available in the DHS program repository, [https://dhsprogram.com/data/].
